# Precision Medicine with 3D Ultrasound

**DOI:** 10.1089/ve.2023.0016

**Published:** 2023-07-13

**Authors:** Ghobad Azizi, Michelle L. Mayo, Lorna L. Ogden, Jessica Farrell, Kele Piper, Carl Malchoff

**Affiliations:** Wilmington Endocrinology, Wilmington, North Carolina, USA.; Thyroid Cytopathology Partners, Austin, Texas, USA.; Massachusetts General Hospital, Boston, Massachusetts, USA.; Carole and Ray Neag Comprehensive Cancer Center, UConn Health, Farmington, Connecticut, USA.

**Keywords:** 3D, ultrasound, thyroid nodule, thyroid cancer

## Abstract

**Introduction:**

A 56-year-old woman was referred for thyroid nodules (TNs) found on a carotid ultrasonography (US). Her laboratories showed a normal thyroid stimulation hormone of 1.530 µIU/mL, normal thyroid hormone levels, and her thyroid antibodies were not elevated. Thyroid 2D US showed an isoechoic solid TN with regular margins measuring 12 × 8 × 10 mm (TR3) in the left thyroid lobe. 3D US demonstrated markedly irregular margins. The nodule volume was 0.52 cm^3^. Based on current American Thyroid Association and American College of Radiology-Thyroid Imaging, Reporting and Data System (ACR-TIRADS) guidelines, her nodule size would not fit the criteria for fine needle aspiration biopsy (FNAB).^1,2^ However, because of the irregular margins seen on 3D US, FNAB was offered along with repeat US after 6 months. After considering her options, she requested FNAB. She underwent effective US guided FNAB of the left TN and the cytopathology report indicated follicular neoplasm Bethesda category IV. Subsequently, she had follow-up US guided FNAB for molecular testing with the Afirma's gene sequencing classifier (GSC). The report showed GSC suspicious with an NRAS mutation, indicating a 50% malignancy risk. She elected to have left hemithyroidectomy. The final surgical pathology report demonstrated a 12-mm follicular carcinoma.

**Materials and Methods:**

In our thyroid clinic, we utilize conventional 2D US and 3D US to evaluate TN for possible FNAB. Laboratory measurements were performed at Labcorp. Informed consent was given by the patient. The 3D image acquisition follows 2D US examination. The first step in 3D US image acquisition is identifying the target nodule utilizing 2D US. Next, the 3D sweep of the target nodule produces a 3D volume data set and observation of 3D-rendered images generated simultaneously from longitudinal, transverse, and coronal views. A 2D US image displays a TN only on one plane in two dimensions, longitudinal or transverse. The saved 3D volume data set can be viewed and manipulated later. We can reconstruct new images from different angles after the study is completed. The 3D image acquisition direction (front to back versus up to down) will create a different display image and volume slice. The examiner can choose the direction of 3D acquisition before 3D sweep. A 2D US image or machine lacks these qualities.

**Discussion:**

This case illuminates recent advances in 3D US imaging and demonstrates that this technology may enhance the value of 2D US in diagnosing malignancy. This technology allows the user to create sequential cross-sectional images through the target nodule. The addition of coronal view to the existing 2D US has been an important contributing factor. Several recent publications have reported that 3D US can improve nodule selection criteria for FNAB.^3–5^ Our clinic has routinely utilized 3D US technology for the past 4 years. We have learned that this new technology can delineate TN borders more clearly. It not only enhances the observation of structures within but also those attached to the thyroid gland. The target nodule can be rotated and viewed from different angles. The margin irregularities of TNs can be viewed with 3D US in small and large nodules equally. We have found that the 3D US shows the irregular margins of malignant TNs to be more pronounced when compared with high-end 2D US systems. In our experience, the vast majority of benign TNs have regular margins on 3D US. Finally, the 3D volume measurement may provide additional information about the size of TNs for longitudinal follow-up of nodules with benign FNAB. The limitations or challenges of using 3D US in general practice include the cost of the ultrasound machine, lack of reimbursement, and the provider's learning curve. Adding 3D/4D technology to current 2D US does provide more detailed information; however, it requires additional time to complete a thyroid US study. 3D US technology might be more suitable for thyroid clinics or endocrine practices with high patient volumes.

**Conclusion:**

We conclude that 3D US can enhance observation of TN margin irregularities and potentially improve nodule selection for FNAB.

No competing financial interests exist.

Runtime of video: 2 hrs 25 mins 12 secs

## Graphical abstract

**Figure:**
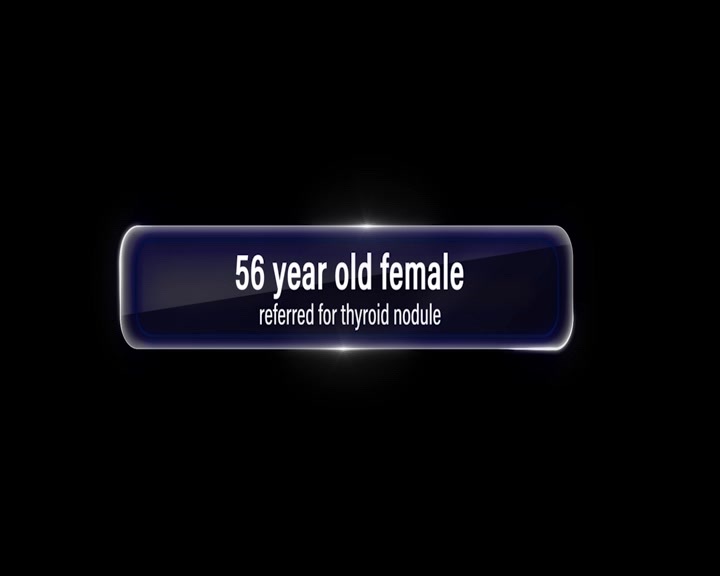

**Figure:**
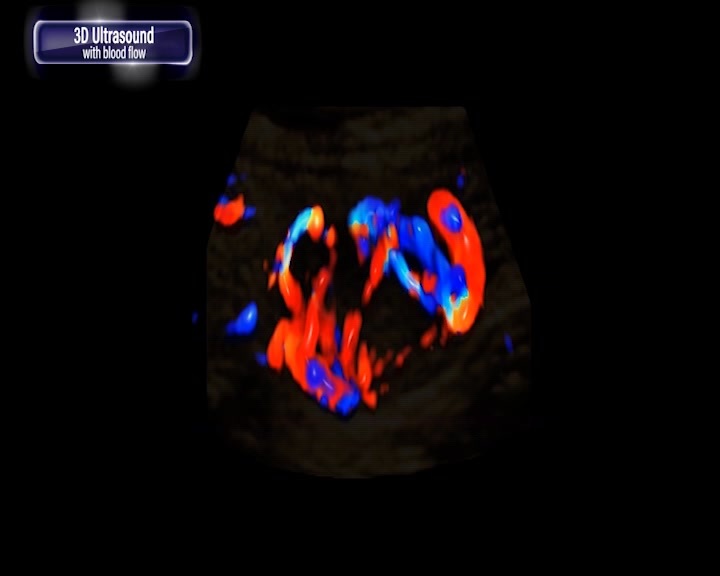

**Figure:**
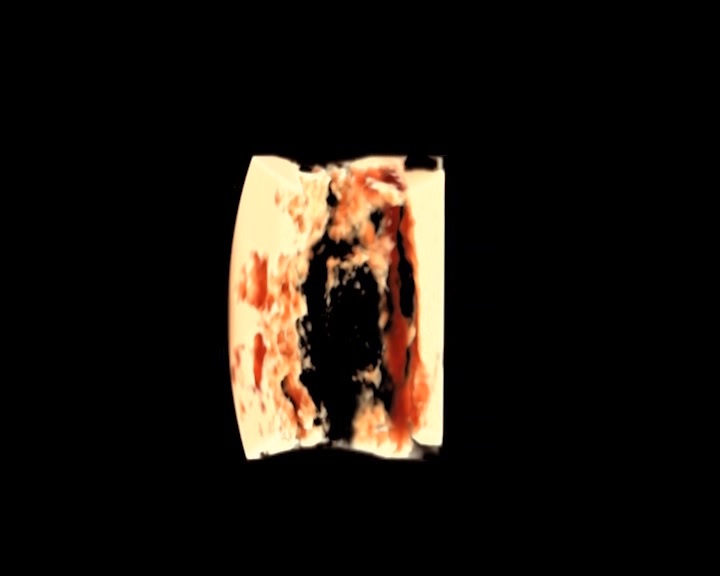

## Video



## Files

**PDF:** 

## Notes

* This article has been updated on June 30, 2023 after first online publication of June 30, 2023 to reflect Open Access, with copyright transferring to the author(s), and a Creative Commons License (CCY-BY) added (http://creativecommons.org/licenses/by/4.0/)
